# A Response to: Commentary: Stabilizing Constructs through Collaboration across Different Research Fields as a Way to Foster the Integrative Approach of the Research Domain Criteria (RDoC) Project

**DOI:** 10.3389/fnhum.2016.00448

**Published:** 2016-08-31

**Authors:** Jacqueline A. Sullivan

**Affiliations:** Department of Philosophy, Rotman Institute of Philosophy, University of Western OntarioLondon, ON, Canada

**Keywords:** construct, DSM, experimental paradigm, psychosocial stress, RDoC

Glannon (2016) argues that mechanistic explanations are reductive and thus not good models for understanding the environmentally situated dynamic causal relationships characteristic of psychopathological phenomena. He rejects my claim that the Research Domain Criteria Project's (RDoC's) success is contingent on the collective stabilization of RDoC constructs (Sullivan, 2016) because he takes construct stabilization to be a requirement applying exclusively to mechanistic explanations. Here, I argue that the dynamic causal relationships Glannon describes require evidential support and construct stabilization has an important role to play in establishing these relationships.

Glannon offers several illustrations of dynamic causal relationships. He explains that acute or chronic psychosocial stress can cause dysregulation of the hypothalamic-pituitary-adrenal (HPA) axis, which increases cortisol levels in the brain. Excess cortisol can contribute to neuronal degeneration in brain areas like prefrontal cortex, which can have deleterious effects on cognition, mood regulation, and motivation.

In support of these dynamic causal relationships, Glannon cites a research study (Höhne et al., 2014) that operationalizes “psychosocial stress” with an experimental paradigm known as *The Trier Social Stress Test (TSST)*. The TSST is “a public speaking task involving a mock job interview and mental arithmetic” (2014, p. 269). In the version of the task used in this study, “participants were given a 10-min preparation time for a presentation about their professional education,” which they had to give “in front of a mixed-gender panel of two judges” who withheld “verbal and non-verbal feedback” (2014, p. 269).

I consulted this research study directly because Glannon does not explain what “psychosocial stress” is. Although the authors of the study indicate the TSST produces psychosocial stress, they define it operationally by means of the TSST. This leaves several questions unanswered: What is psychosocial stress? Are there different types? Do all types activate the same cascades of events (e.g., psychological/cognitive, cellular, molecular)? What types of phenomena are produced using the TSST? How do these phenomena compare with phenomena produced by other psychosocial stress-inducing experimental paradigms?

Two exemplary meta-analyses contain some responses to these questions. Dickerson and Kemeny, for example, focused on 208 studies investigating “the effects of psychosocial stressors on cortisol activation” (Dickerson and Kemeny, 2004, p. 355). Their motivation was that cortisol changes are not “uniformly triggered” across experimental paradigms used to induce psychosocial stress, so they wanted to determine what features set some experimental paradigms apart. They claim those experimental paradigms accompanied by the greatest increases in cortisol placed subjects in situations where they were “in danger of negative evaluation of important and valued aspects of [themselves] by others” (“social-evaluative threat”) and were unable to control situation outcome (“outcome uncontrollability”) (2004, p. 377).

Dickerson and Kemeny's study is suggestive that not all experimental paradigms used to study the relationship between psychosocial stress and cortisol responses are equivalent. Insofar as task demands or type of stressors differ across experimental paradigms, it is likely that different cascades of events (e.g., psychological/cognitive, neurophysiological) are triggered when subjects are trained in them. Dickerson and Kemeny also indicate that if we use the word “stress in a vague and diffuse way” these differences may be obscured and “prevent[] focused research on specific kinds of threats that can affect health-relevant physiological systems” (Dickerson and Kemeny, 2004, p. 377).

A second meta-analysis undertaken by Frisch and colleagues addresses the question of what phenomena the TSST measures. They regard the fact that the “scope of the TSST is limited to inducing […] social-evaluative threat” (2015, p. 12) as a weakness. They also claim that because the TSST contains “two-stress-inducing-elements” namely, social evaluation and uncontrollability, it is difficult to determine whether each element has “independent effects” downstream (e.g., on psychological/cognitive processes or neurophysiological processes). Despite these weaknesses they assert that “the TSST protocol is highly standardized” and this “high degree of standardization allows for comparisons between different studies in one field of research” which “facilitates the integration of these findings in reviews and meta-analysis” and allows for “the replication and extension of previous studies” (Frisch et al., 2015, p. 12). At best, what can be concluded on the basis of Frisch and colleagues' analysis is that the TSST may be a valuable experimental paradigm for studying the relationship between social-evaluative threats and neurophysiological stress responses.

These two meta-analyses taken in combination lend support to my argument that establishing causal claims—whether dynamic or mechanistic—requires the collective stabilization of the terms those claims contain and phenomena to which they make reference. If RDoC is to promote precision medicine in the clinic and the RDoC matrix includes terms that do not have explicit shared definitions or terms that are operationalized differently across research studies and investigators, then how can clinicians learn how to effectively deploy these terms to determine which causal relationships are relevant and which therapeutic interventions will be most effective for treating their patients?

These two studies also point to strategies that may facilitate progress with respect to psychological constructs in general, and that may promote progress with respect to RDoC constructs in particular. Specifically, investigators need to be clear about how they are generally defining terms and what phenomena their experimental paradigms may be used to produce, detect, and measure. They also need to strike a balance between implementing measures that will facilitate the replicability of their results (Sochat et al., 2016) while not preventing the possibility of novel discoveries.

On a final note, DSM-5 categories may be understood as “diagnostic kinds” (Tabb, 2015); DSM authors have taken measures to stabilize the use and meanings of DSM terms across clinicians (Haslam, 2013). RDoC constructs lack this kind of stability– they are “research kinds”, which are heuristics subject to change in light of new discoveries (Bechtel and Richardson, 1993/2010; Cuthbert and Insel, 2013). If precision medicine is RDoC's aim, application of terms designating “research kinds” in clinical contexts will require at least a minimal degree of stability, otherwise clinicians will potentially run the risk of misdiagnosing their patients and providing them with inadequate medical care. I only am advocating for changes to investigative, conceptual and integrative practices that may facilitate the precision medicine goal.

## Author contributions

The author confirms being the sole contributor of this work and approved it for publication.

### Conflict of interest statement

The author declares that the research was conducted in the absence of any commercial or financial relationships that could be construed as a potential conflict of interest.
